# Sustainable Cellulose Nanofibril−Stabilized Pickering Emulsions for Fresh Egg Preservation

**DOI:** 10.3390/nano15070515

**Published:** 2025-03-29

**Authors:** Hao Li, Lei Zhang, Mei Cui, Renliang Huang, Rongxin Su

**Affiliations:** 1 State Key Laboratory of Chemical Engineering, Tianjin Key Laboratory of Membrane Science and Desalination Technology, School of Chemical Engineering and Technology, Tianjin University, Tianjin 300072, Chinasurx@tju.edu.cn (R.S.); 2 Tianjin Key Laboratory for Marine Environmental Research and Service, School of Marine Science and Technology, Tianjin University, Tianjin 300072, China; 3 Zhejiang Institute of Tianjin University, Ningbo 315201, China

**Keywords:** nanocellulose, cellulose nanofibrils, Pickering emulsion, edible coatings, egg preservation

## Abstract

Eggs are perishable mainly due to moisture loss and bacterial contamination. Herein, we developed an efficient and sustainable coating emulsion for egg preservation, which is primarily composed of cellulose nanofibrils (CNFs), coconut oil (CO), cinnamaldehyde, and sophorolipids. The effects of weight ratios of CO to CNFs on emulsion stability and the crucial properties of the resulting coatings were systematically investigated. Under the optimal weight ratio of 1:1, the emulsion demonstrated excellent stability, with a zeta potential of −50.86 mV, and the coating exhibited a low water vapor transmission rate of 18.19 g mm m^−2^ day^−1^. Moreover, the addition of cinnamaldehyde and sophorolipids enhanced the antibacterial properties of the coating and the wettability of the emulsion on eggshells. After 4 weeks of storage at 25 °C, the eggs coated with the developed emulsion deteriorated from AA to A grade, while those coated with mineral oil and the uncoated eggs deteriorated to B grade. Overall, this work presents a promising, sustainable, easily scalable, and highly efficient technique for fresh egg preservation, which holds great potential for practical applications in the egg storage industry.

## 1. Introduction

According to the Food and Agriculture Organization of the United Nations, 733 million people faced hunger in 2023 [1]. In this context, eggs serve as a highly nutritious food source that helps address dietary deficiencies. Due to their similar amino acid compositions to those of human body, egg proteins are highly bioavailable and can be easily absorbed [2]. Moreover, eggs are rich in vitamin A, with content levels twelve times higher than vegetables like carrots [3]. Additionally, choline in eggs is an essential micronutrient for human beings, supporting cell division, growth, and membrane signaling [4]. However, eggs are perishable, resulting in a short shelf life and huge food loss. The internal quality of eggs deteriorates rapidly due to the loss of moisture and carbon dioxide through ~10,000 tiny pores on the eggshell [5]. Besides, eggs are susceptible to *Salmonella enteritidis* which easily leads to food poisoning with symptoms like diarrhea, vomiting, and fever [6].

Currently, the processing of eggs before retail and consumption mainly includes washing, ultraviolet sterilization and coating with mineral oil [7]. While washing effectively eliminates contaminants from eggshells, the cuticle layer is probably damaged, making the preservation difficult. As a consequence, mineral oil coating is commercially applied to preserve eggs. However, a large amount of mineral oil is required to achieve suitable barrier performance due to its poor film-forming capability, inevitably leading to inferior appearance. In addition, owing to the lack of antibacterial properties, mineral oil-coated eggs pose a significant risk of microbial contamination [8]. Moreover, the extensive use of petroleum-based mineral oil is not a green and sustainable approach [9]. To address these challenges, proteins, polysaccharides, and lipids are generally used for coating manufacturing due to their edibility and renewability [10]. For example, Caner and Yüceer [11] developed a whey protein concentrate-based coating, which effectively extended eggs’ shelf life, allowing them to maintain A grade even after 5 weeks of storage at 24 °C. However, proteins are recognized as common allergens and their widespread use is restrained [12,13]. Chitosan and edible oils were also demonstrated to be effective to preserve internal quality of eggs [14,15]. Nonetheless, chitosan and other polysaccharide-based coatings exhibited poor water resistance [16], while oil-based coatings are easily oxidized, and neither of them demonstrated adequate antibacterial activity [17,18]. Therefore, it is imperative to develop a multi-functional, efficient, and sustainable coating for egg preservation [19].

**Figure 1:**
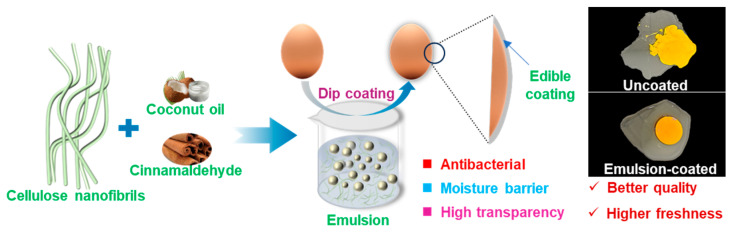
Schematic diagram illustrating the fabrication of CNF-based Pickering emulsion and its application in egg preservation.

Cellulose nanofibrils (CNFs) are renewable and biodegradable nanomaterials, which exhibit excellent film-forming capability and abundant surface hydroxyls, allowing them to form flexible and robust films with superior gas barrier performance [20]. In addition, the hydrophilic hydroxyl groups enable them the ability to stabilize oil−water interface to form Pickering emulsions [21,22]. Moreover, the entangled network structure of CNFs also prevents oil droplet coalescence, thereby enhancing emulsion stability [23]. Coconut oil (CO) is a natural lipid with high stability and saturated fat content, confirming a number of health benefits such as cholesterol-lowering, anti-cancer, and anti-inflammatory properties [24]. Currently, CO has gained worldwide popularity due to its commercial application in food, cosmetics, and medication. Cinnamaldehyde is a natural antimicrobial compound, which is listed as “Generally Recognized as Safe” by the Food and Drug Administration [14] and has been widely used for food preservation. Since dip-coating was employed for the fabrication coatings on eggs, the addition of sophorolipid is necessary to enhance wettability on eggs [25]. To date, the incorporation of CNFs, CO, cinnamaldehyde, and sophorolipids for egg preservation (Figure 1) remains unexplored. This study represents a pioneering approach in the incorporation of CO into CNF-based composites for egg preservation. While leveraging CO’s superior oxidative stability—attributable to its high saturated fatty acid content that resists peroxidation under ambient conditions [26]—the coating adhering to eggshells will not cause the potential risk of excessive saturated fatty acid intake [24]. Furthermore, the critical surface tension of eggshells was investigated, which helps mitigate the risk of coating delamination due to interfacial incompatibility. This investigation provides a theoretical foundation for the incorporation of surfactants in egg preservation coatings, ensuring the correct use of surfactants to enhance coating performance [27].

The weight ratio of CO to CNFs was optimized to improve the stability of the emulsion, the mechanical properties, water vapor barrier capability, and transparency of the emulsion-derived coatings. Under the optimal weight ratio of CO to CNFs, the antibacterial performance of the coating was validated and the surface tension of the emulsion was modulated to enhance wettability on eggshells. Finally, the efficacy of the coating in maintaining egg freshness and reducing weight loss was assessed at 25 °C and 60% relative humidity (RH). The stability of the emulsion was evaluated using a Zetasizer Nano ZS to measure the average particle size and zeta potential. The properties of the emulsion-derived coatings were assessed using a universal testing machine, permeability cup, and UV-visible spectrophotometer. The morphology of cellulose nanofibers (CNFs), eggshells, and the coatings was observed through scanning electron microscopy (SEM) and transmission electron microscopy (TEM). Surface tension and contact angle measurements were conducted using a OneAttension Theta Lite contact angle meter.

## 2. Materials and Methods

### 2.1. Materials

Pulp was purchased from Dalian Yangrun Trading Co., Ltd. (Dalian, China). All reagents involved in this study are listed in the table below (Table 1). All chemicals were used without any further purification. Gas chromatography is represented by GC, the analytical reagent is represented by AR, and the biological reagent is represented by BR. Fresh eggs (two days old) with no visual defects, uniform color, and a weight range of 45–65 g were purchased from Tianjin Guangyuan Livestock and Poultry Breeding Co., Ltd., (Tianjin, China).

### 2.2. Preparation of Coating Emulsions and Films

CNFs were prepared from softwood pulp via TEMPO-mediated oxidation (pH 10.0, 7 mmol NaClO/g cellulose) and subsequent mechanical disintegration using a high-pressure homogenizer (1000 bar, six passes) as described in our prior work [28]. To prepare Pickering emulsions, CNFs, CO, glycerol, sophorolipids, and cinnamaldehyde (Table 2) were homogenized using a juicer (JYL-Y921, Jinan, China) at 15,000 rpm for 2 min. After degassing, the emulsions were poured into plastic Petri dishes and dried in the oven at 25 °C for 24 h to obtain composite films.

### 2.3. Characterization

The CNF suspension (0.3 wt%) was modified to pH 3.0 using 0.1 M HCl, followed by NaOH titration (0.1 M, 0.1 mL/min) via a peristaltic pump. Real-time conductivity monitoring (DDS-307A, China) continued until values exceeded the initial baseline, and the charge content of CNFs was calculated by Equation (1) [25].

(1) $$C_{-COOH}=\frac{(V_{2}-V_{1})\times c}{m}$$

where *C_-COOH_* is the content of carboxyl group on CNFs (mmol/g). *V*_1_ is the volume of standard sodium hydroxide solution consumed at the first equivocal point (L); *V*_2_ is the volume of standard sodium hydroxide solution consumed at the second equivocal point (L); *c* is the concentration of sodium hydroxide standard solution (mmol/L); *m* is the dry weight of CNFs (g). To observe the size and morphology of CNFs, a 0.001 wt% CNF dispersion was ultrasonicated for 20 min in an ice bath to prevent overheating. Subsequently, 6 μL of the suspension was deposited onto a copper mesh, air-dried at room temperature, and stained with 8 μL of 1 wt% phosphotungstic acid solution for 90 s. Excess stain was blotted using filter paper, and the CNFs morphology was characterized by transmission electron microscopy (TEM). The morphology of the coating and eggshell surface were observed using a scanning electron microscopy (SEM, Regulus 8100, Hitachi, Japan) at an accelerating voltage of 5 kV, with a thin layer of gold sprayed on before observation [26].

### 2.4. Measurement of Emulsion Properties

The average particle size and zeta potential of the coating emulsion were measured using a Zetasizer Nano ZS (Malvern Instruments Ltd., Malvern, UK). The emulsions were diluted 40 times using ultrapure water to achieve a particle concentration within the instrument’s optimal detection range (0.01–0.1 wt%), and then 0.8 mL of the diluted samples were added into a sample tank, and the average particle size and zeta potential were detected separately after 2 min of equilibrium at 25 °C [18,27].

The turbidity of the coating emulsion was measured according to the method reported by Pan et al. [29]. The absorbance of the emulsion was measured at 600 nm with a UV spectrophotometer (TU-1810PC, Beijing, China) [28] and the turbidity was calculated according to following Equation (2):

(2) $$T=\frac{2.302A\times V}{I}$$

where *A* is the absorbance of the diluted coating emulsion at 600 nm; *V* is the dilution factor; *I* is the optical path difference (0.01 m).

To test the static stability, the coating emulsions were placed in dark at room temperature for 1 month. Pictures were captured regularly to observe whether there is phase separation or flocculation.

### 2.5. Mechanical and Water Barrier Properties of the Emulsion-Derived Films

The mechanical properties of derived films were evaluated according to Chinese standard GB/T 1040.3-2006 [30]. Before measurement, the films were conditioned in a chamber (CTHI-150B, STIK, Shanghai, China) at 25 °C and 60% RH for 12 h. The films were cut into strips (1 cm × 6 cm), and the thickness was measured using a micrometer screw. The mechanical properties were then tested using a universal testing machine (AGS-X-10kN, Shimadzu Corporation, Kyoto, Japan) at a tensile speed of 5 mm/min [29].

The water vapor transmission rate (WVTR) of the coating was evaluated according to GB/T 1037-2021 [31]. Briefly, the circular sized films were prepared as test specimens and the permeability cup was filled with silica gel, leaving a small air gap between the specimen and silica gel. The permeability cup was kept at 25 °C and 60% RH for 12 h to measure the increase in cup weight. In addition, the light transmittance of the films was measured by the UV-visible spectrophotometer at the range of 300–700 nm [32].

### 2.6. Antibacterial Performance of the Emulsion-Derived Films

The antibacterial performance of the films was evaluated using *Escherichia coli* (*E. coli*). The bacterial suspension without the films was used as the control, and in the experimental group, the bacteria were co-cultured with the films at 37 °C for 4 h. Subsequently, the bacteria solution was diluted using PBS solution and 100 μL of the resulting mixture was spread onto LB plates and then incubated at 37 °C for 24 h for observation and counting.

### 2.7. Wettability of the Coating Emulsion

The surface tension of the coating emulsion was measured using the pendant drop method with the OneAttension Theta Lite contact angle meter (Biolin Scientific, Gothenburg, Sweden). Specifically, 4 μL droplets of the emulsion were placed on a precision stainless steel needle (inner diameter 0.5 mm) at room temperature. The droplet shape was analyzed through high-speed imaging, and the surface tension was calculated applying the Young-Laplace equation with the Bashforth-Adams numerical approximation. The critical surface tension (*γ*_c_) of the eggshell was obtained according to the method reported by Deng et al. [32]. The contact angles (CA) of water, diiodomethane, and ethylene glycol on the eggshells were measured via the sessile drop method. For each liquid, 4 μL droplets were deposited on the eggshell surface. A Zisman plot was constructed by correlating cos (θ) (*y*-axis) with liquid surface tension (*γ*_L_, *x*-axis), and *γ*_c_ was extrapolated from the linear regression fit at cos (θ) = 1.

### 2.8. Evaluation of Egg Quality During Storage

Eggs were washed with 40 °C hot water [33] to remove feces from the surface and exposed to ultraviolet light for 30 s. Then, the eggs were treated through three different methods: uncoated, coated with mineral oil, and coated with emulsion. To fabricate coating, the eggs for mineral oil coating were spray-coated for 10 s using the egg coating machine, while the eggs for emulsion coating were immersed in the coating emulsion for 10 s. After drying at room temperature, uncoated, mineral oil-coated and emulsion-coated eggs were stored at 25 °C, 60% RH to assess the preservation efficacy.

The sensory of albumen and yolk was observed by cracking the eggshell and spreading the egg content on the glass plate. The weight loss (%) and the Haugh unit of eggs were calculated using Equations (3) and (4), respectively. The pH of albumen was evaluated using a pH meter (MP522, Shanghai Lida Instrument Factory, Shanghai, China). The yolk index of eggs was calculated by Equation (5).

(3) $$Weight\;loss=\frac{initial\;weight-present\;weight}{initial\;weight}\times 100\%$$

(4) $$Haugh\;unit=100\times log(h+7.57-1.7\times W^{0.37})$$

(5) $$Yolk\;index=\frac{The\;height\;of\;yolk}{The\;width\;of\;yolk}$$

where *W* is the weight of the whole egg (g), *h* is the thick albumen height (mm).

## 3. Results

### 3.1. Characterization of the CNFs

The prepared negatively charged CNFs (1.26 mmol/g, Figure S1) revealed a fibril morphology of a length of 300–700 nm and a width of 4–6 nm (Figure S2), in line with previous report [28]. Due to their high aspect ratios (50–175), CNFs tend to form an entangled network structure, creating gel-like suspensions that prevent oil droplet coalescence and thus enhance emulsion stability. Once dried, the nanofibrils will organize into a compact layered network structure that contribute to superior gas barrier properties [34].

### 3.2. Optimization of the Weight Ratio of Coconut Oil to CNFs

#### 3.2.1. Stability of the Emulsions

**Figure 2:**
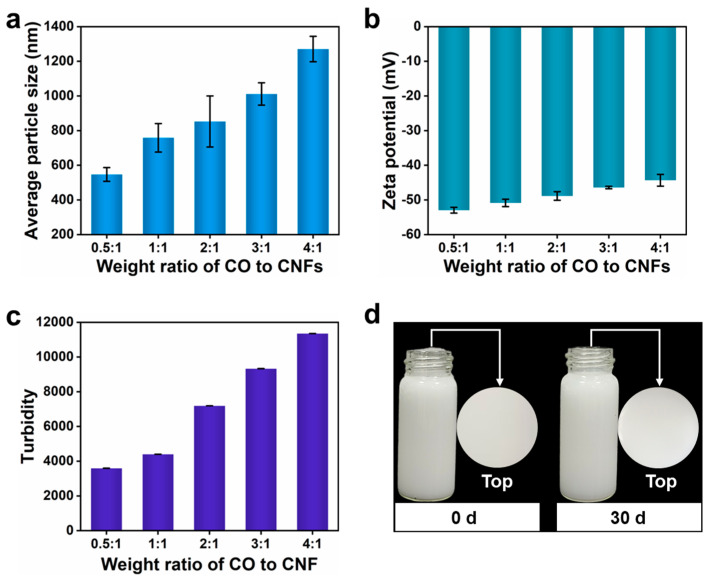
Effects of weight ratio of CO to CNFs on the average droplet size (**a**), zeta potential (**b**), and turbidity (**c**) of coating emulsion. Photographs of the coating emulsion prepared with a 1:1 weight ratio of CO to CNFs at day 0 and 30 (**d**).

Figure S3 presents the emulsions prepared using CNF suspensions and CO with the weight ratios of CO to CNFs ranging from 0.5:1 to 4:1. Once emulsified, all oil was dispersed as droplets, indicating the formation of the Pickering emulsions. To further assess their stability, the critical parameters (average particle size and zeta potential) were systematically investigated [35,36]. As shown in Figure 2a,b, the average particle size and zeta potential of the emulsion droplets were significantly affected by the weight ratio of CO to CNFs. As the weight ratio of CO to CNFs increased from 0.5:1 to 4:1, the average particle size increased from 547.1 nm to 1270 nm, and the zeta potential increased from −52.97 mV to −44.32 mV. This was probably attributed to that fewer CNFs can partition at the oil−water interface to stabilize emulsion droplets with the increasing of the CO content, weakening the steric effect and electrostatic effect between oil droplets [37]. As a result, the average particle size increased and the absolute zeta potential decreased. Meanwhile, the turbidity of the emulsion dramatically increased from 3594 to 11,353 (Figure 2c) as a result of the increase of emulsion droplet size and concentration [36]. After storage for one month at room temperature, emulsions with a weight ratio of >1:1 released pure oil at the top layer, meaning the occurrence of coalescence and a weak stability (Figure S3). In contrast, emulsions with weight ratios of 0.5:1 and 1:1 demonstrated superior stability without any visible coalescence (Figure 2d).

#### 3.2.2. Mechanical and Water Barrier Properties of the Films from Emulsions

**Figure 3:**
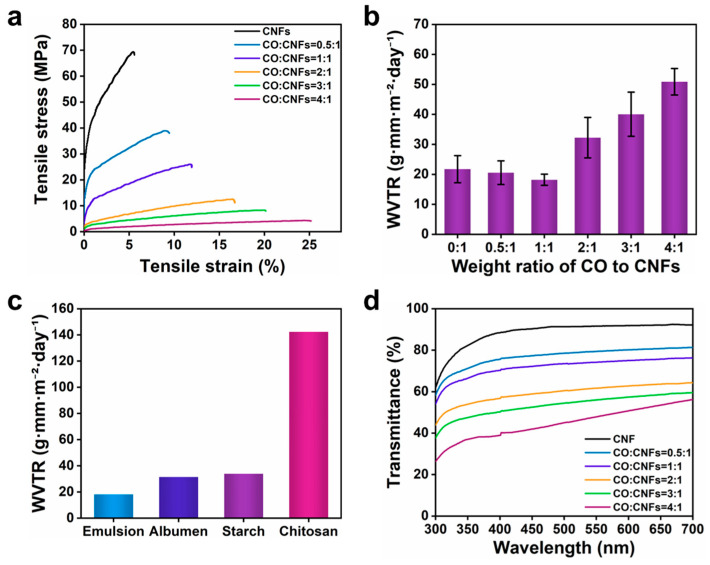
Effects of the weight ratio of CO to CNFs on the mechanical performance (**a**), WVTR (**b**), and transparency (**d**) of the derived films. WVTR (**c**) of our coating compared to common biobased coatings [41,42,43].

Mechanical robustness and flexibility are essential for coatings to withstand bending, twisting, and folding during handling and storage, which significantly affected their durability and integrity. As shown in Figure 3a and Figure S4, pure CNF film exhibited the highest tensile strength (74.2 MPa) but the lowest elongation at break (6.1%) due to the strong hydrogen bonding between the hydroxyl groups. With the increasing of CO content, the tensile strength reduced to 4.5 MPa while the elongation at break increased to 22.7%. The reduction in tensile strength was ascribed to the weak interfacial adhesion between CO and CNFs [38]. It is worth noting that, even when the weight ratio of CO to CNFs reaches 2:1, the films exhibited better mechanical properties (11.3 MPa, 15.9%) in comparison with the hyaluronic acid-curcumin-cellulose nanofiber composite films (7.81 MPa, 9.46%) prepared by Fan et al. [39] for egg preservation.

Moisture loss primarily results in the weight loss of eggs, making it crucial to enhance the water vapor barrier properties of coatings for egg preservation [40]. As shown in Figure 3b, pure CNF films revealed a water vapor transmission rate (WVTR) of 21.7 g mm m^−2^ day^−1^. As the weight ratio of CO to CNFs increased to 1:1, the WVTR of the derived films reduced to 18.2 g mm m^−2^ day^−1^, which was smaller than that of common biobased coatings used for egg preservation, including starch [41], albumen [42], and chitosan [43] (Figure 3c). However, as the weight ratio continued to increase to 4:1, the WVTR dramatically increased to 50.9 g mm m^−2^ day^−1^. At low weight ratios (≤1:1), the addition of CO improved the hydrophobicity of hydrophilic CNF films, leading to an enhanced water barrier performance. However, the CO cannot be uniformly dispersed in the CNF matrix at higher weight ratios, producing oil aggregates, micropores, holes, and weakening the structural integrity that reduced water barrier property.

Moreover, considering that consumers have a preference for eggs with visually assessable appearances, the optical properties of the films were evaluated. As shown in Figure 3d, the CNF films exhibited high transparency (~90%) ranging from 400 nm to 700 nm due to nanosized diameter of CNFs (4–6 nm). With the increasing of CO, the transmittance of the films decreased due to light scattering caused by oil particles in the CNF matrix [44]. The coating with a weight ratio of 1:1 maintained a high transmittance (exceeding 70%) within the wavelength range of 400–700 nm. This property enables consumers to clearly view and accurately evaluate the appearance of the eggs coated with this emulsion.

In general, considering the emulsion stability, as well as the mechanical, optical, and barrier properties of the derived films, the optimal weight ratio of CO to CNFs was chosen to be 1:1 for the following experiments.

### 3.3. Antibacterial Performance of the Coatings

**Figure 4:**
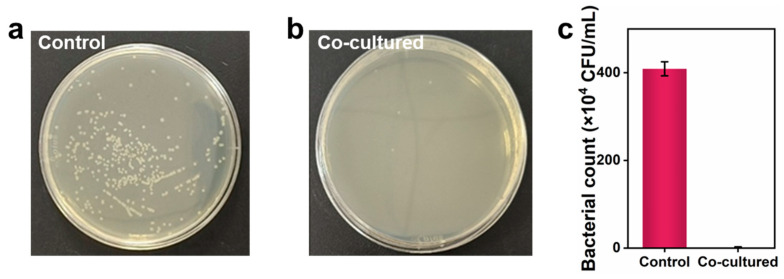
Photographs of bacterial colonies formed by (**a**) *E.coli* and (**b**) *E.coli* treated with our coating. Statistics of the bacterial counts (**c**).

To confer antibacterial activity to the coatings, cinnamaldehyde was introduced into the emulsions as natural active compounds. To assess the antibacterial performance of our coating, *E.coli* was co-cultured with the test specimen. As depicted in Figure 4, the control group exhibited a bacterial concentration of ~4 × 10^6^ CFU/mL, whereas our coating showed no bacterial titers. As a consequence, the excellent antibacterial activity would mitigate bacterial contamination during egg storage and extend shelf life, with enhanced egg quality and safety.

### 3.4. Interaction of Coating with Eggshell Surface

**Figure 5:**
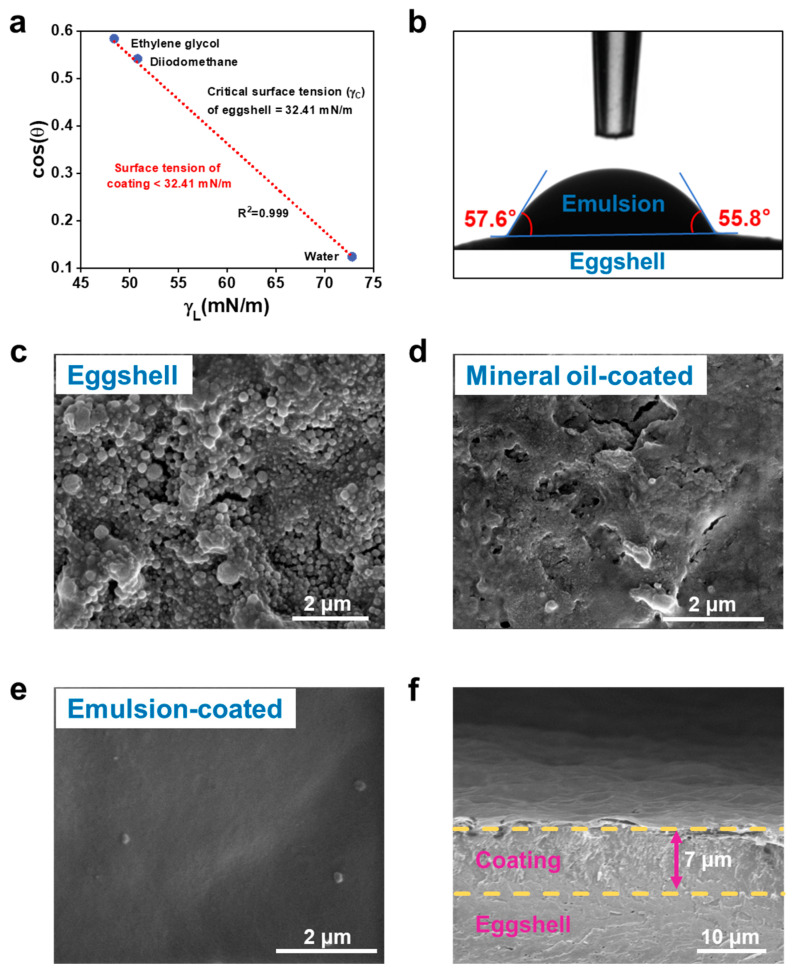
The critical surface tension of the eggshell (**a**). The contact angle of the emulsion on the eggshell (**b**). The shell surface morphology of uncoated eggs (**c**), mineral oil-coated eggs (**d**), emulsion-coated eggs (**e**), and the cross-section images of the emulsion-coated eggshell (**f**).

In this work, dip-coating was employed for the fabrication coating on the surface of eggs. We first investigated the affinity of the coating emulsion to the eggshell by evaluating the surface tension of coating emulsion and surface free energy of eggshell. As depicted in Figure 5a, the eggshell showed a *γ*_c_ of approximately 32.41 mN/m according to the Zisman plot [45]. The addition of sophorolipids reduced the surface tension of the coating emulsion from 49.97 mN/m to 31.97 mN/m (Figure S5), ensuring the emulsion an enhanced wettability on the eggshell with a contact angle of 56.7° (Figure 5b). To investigate the thickness and morphology of the coating, SEM was used to view the eggshell surface of before and after coating. As shown in Figure 5c, the shell of uncoated eggs exhibited a roughness and porous structure. The surface became smoother and the number of pores of eggshell decreased after being coated with mineral oil (Figure 5d). Compared to mineral oil, our emulsion exhibited a better film-forming ability and produced a visible smooth layer that covered the eggshell with a thickness of about 7 μm (Figure 5e,f). This conformal coating with no visible pores illustrated outstanding adhesion on the eggshell (Figure 5f) and thus would serve as a barrier layer that against gas and water vapor, confirming its great potential for egg preservation.

### 3.5. Effects of Coating Emulsion on Egg Quality and Shelf Life

#### 3.5.1. Sensory Evaluation

**Figure 6:**
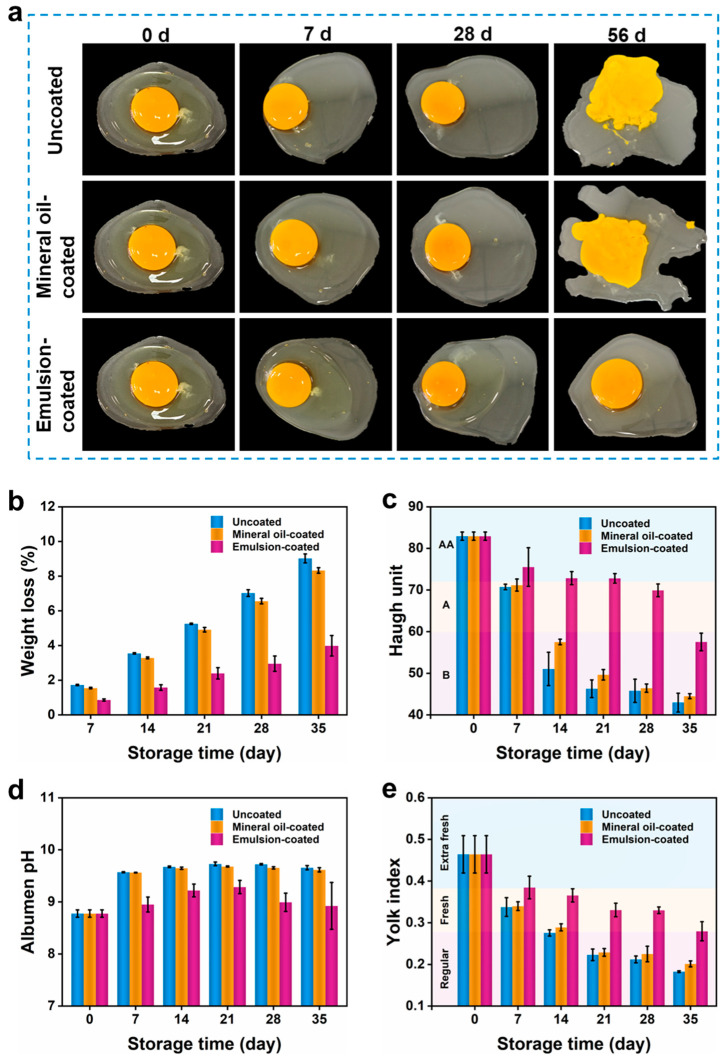
Time-lapse photographs of coated and uncoated eggs (**a**). Weight loss (**b**), Haugh unit (**c**), albumen pH (**d**), and yolk index (**e**) of eggs during storage at 25 °C/60% RH.

The sensory changes in egg white and yolk of coated and uncoated eggs were depicted in Figure 6a. As storage time prolonged, the egg’s odor gradually faded, the egg white became thinner, and the yolk lost its elasticity. The egg white of uncoated eggs exhibited watery behavior within one week due to protein hydration, while eggs coated with mineral oil and our coating only displayed similar same behavior in the fourth and eighth weeks, respectively. Moreover, the uncoated and mineral-coated eggs exhibited yolk dispersal after eight weeks, whereas the eggs coated with our coating presented intact yolk.

#### 3.5.2. Weight Loss

The weight loss of eggs was mainly ascribed to the water loss from the egg white [42]. During storage, water vapor inside the egg left the egg through the pores on the eggshell, resulting a significant decrease in egg weight (9.02% after 35-day storage) (Figure 6b), while for mineral oil-coated eggs, the weight loss after 35 days (8.33%) was slightly lower than that of uncoated eggs. In contrast, the emulsion-coated eggs demonstrated a minimal weight loss of 3.99% after 35 days which was even lower than that observed in the uncoated and mineral oil-coated eggs at day 21, confirming its superior capacity to mitigate water loss. Moreover, this emulsion coating significantly reduced the weight loss by >50% across the whole storage period, surpassing the existing egg protein coatings [42].

#### 3.5.3. Haugh Unit

To quantitatively assess egg internal quality and freshness, we monitored their Haugh unit during storage. Eggs are typically categorized into four grades to determine if the egg quality are high enough to be sold according to their Haugh unit: AA grade (Haugh unit ≥ 72), A grade (60 ≤ Haugh unit < 72), B grade (30 ≤ Haugh unit < 60), and C grade (Haugh unit < 30) [46]. Figure 6c exhibits the Haugh unit of uncoated and coated eggs during 8 weeks of storage. While fresh eggs were AA grade (Haugh unit = 83), uncoated and mineral oil-coated eggs rapidly dropped below AA grade after one week and continued to drop to B grade in the second week. Conversely, eggs with our coating maintained AA grade after 3-week storage and their Haugh unit in the fourth week was comparable to that of the uncoated eggs in the first week. Even after 5 weeks, the Haugh unit of emulsion-coated eggs remained above 55, which was higher than that of uncoated and mineral oil-coated eggs in the second week. This was attributed to that our coating mitigated carbon dioxide loss and microbial contamination, thereby restraining protease activity, protein hydrolysis, and protein degradation. Overall, these results demonstrated that our coating was highly effective in preserving egg quality and freshness, ensuring that the eggs maintain AA grade for up to 3 weeks.

#### 3.5.4. Albumen pH

Typically, the loss of carbon dioxide through the pores on the eggshell and the disintegration of egg white resulted in an increase of albumen pH [42], displaying egg freshness. Notably, the original pH of the two days old eggs was 8.78 (Figure 6d) which was higher than that of freshly laid eggs (7.6–8.5). As depicted in Figure 6d, the albumen pH of uncoated and mineral oil-coated eggs rapidly increased to 9.57 and 9.56, respectively, in the first week, and remained above 9.6 during whole storage period. While for eggs coated with our coating, the pH increased slightly to 8.95 in the first week and revealed a maximum pH of 9.29 in the third week, indicating better freshness in comparison with uncoated and mineral oil-coated eggs. In the last two weeks, the albumen pH of emulsion-coated eggs reduced to 8.92 since the coating minimized carbon dioxide loss [47]. It can be concluded that our coating provides an excellent barrier against carbon dioxide and water vapor during storage, effectively preserving egg freshness.

#### 3.5.5. Yolk Index

The yolk index indicates the strength of the yolk vitelline membrane and the spherical shape of the yolk, which can be also used to qualitatively evaluate egg freshness: Extra fresh (yolk index > 0.38), Fresh (0.28 ≤ yolk index ≤ 0.38), and Regular (yolk index < 0.28) [14,42]. Figure 6e represents the yolk index of uncoated and coated eggs during 5-week storage. The yolk index of uncoated eggs reduced from 0.46 to 0.27 in the second week, indicating a “Regular” category. This was attributed to the hydration of albumen that changed the osmotic pressure and destroyed its structural integrity, thereby weakening its support for yolk and reducing the yolk index. Moreover, when the yolk index falls below a critical threshold, yolk may disperse (Figure 6a) and adhere to the eggshell [48]. While for mineral oil-coated eggs, the yolk index remained above 0.28 in the first two weeks and decreased to 0.23 in the third week, demonstrating a better freshness. On the contrary, the yolk index of emulsion-coated eggs in the second week was measured to be 0.38 (Extra fresh) and sustained above 0.28 that belonged to Fresh category even after five weeks, confirming the high efficacy of our coating on egg freshness preservation.

## 4. Conclusions

In summary, a sustainable Pickering emulsion mainly composed of sustainable cellulose nanofibrils, coconut oil, cinnamaldehyde, and sophorolipids was successfully developed for egg preservation. Under the 1:1 weight ratio of CO to CNFs, the emulsion demonstrated excellent stability (without any visible coalescence during 1 month) and the coating fabricated from it exhibited outstanding water vapor barrier performance (18.19 g mm m^−2^ day^−1^), mechanical properties (tensile strength 26.2 MPa, elongation at break 12.7%), and transparency (exceeding 70%). The incorporation of sophorolipids and cinnamaldehyde significantly enhanced the wettability of the emulsion on eggshells and the antibacterial activity of the coatings. By employing the dip-coating method, conformal micron-thickness coatings could be facilely fabricated on eggshells. When stored at 25 °C/60% RH, compared with uncoated eggs and those coated with commercial mineral oil, the eggs coated with the developed emulsion showed an extended shelf life (over three weeks), reduced weight loss (by >50%), better-maintained internal quality, and higher freshness (AA grade for 3 weeks). Notably, the synthesis process of this coating is scalable. All the starting materials are recognized as safe, and the dip-coating technology is commercially accessible. These advantages render it a promising candidate for reducing egg waste, thereby contributing to enhancing food security and nutrition.

## Figures and Tables

**Table 1:** Reagent, specification, and manufacturer thereof.

Reagent	Specification	Manufacturer
2,2,6,6-tetramethylpiperidine-1-oxyl radical (TEMPO)	≥95%, GC	Aladdin, Shanghai, China
Sodium bromide (NaBr)	≥99%, AR	Aladdin, Shanghai, China
Sodium hydroxide (NaOH)	≥96%, AR	Aladdin, Shanghai, China
Sodium Hypochlorite (NaClO)	Effective chlorine ≥5.0%	Aladdin, Shanghai, China
Glycerol	≥99%, GC	Aladdin, Shanghai, China
Phosphate buffer saline buffer solution (PBS)	pH 6.8	Aladdin, Shanghai, China
Food-grade coconut oil (CO)	95%	Tianjin Hope Technology Co., Ltd., Tianjin, China
Sophorolipids	BR	NJDULY, Nanjing, China
Cinnamaldehyde	98%	Tianjin Hope Technology Co., Ltd., Tianjin, China
Nutrient agar	BR	Beijing Aobostar Biotechnology, Beijing, China

**Table 2:** The amounts of raw materials for five proportions of Pickering emulsions.

CO/CNFs	Mass of CO (g)	Mass of CNFs (g, Dry Weight)	Mass of Glycerol (g)	Mass of Cinnamaldehyde (g)	Mass of Sophorolipids (g)
0.5:1	0.5	1	0.45	0.1	0.6
1:1	1	1	0.6	0.1	0.6
2:1	2	1	0.9	0.1	0.6
3:1	3	1	1.2	0.1	0.6
4:1	4	1	1.5	0.1	0.6

## Data Availability

The data presented in the following study are available from the first author upon request.

## Supplementary Materials

The following supporting information can be downloaded at: https://www.mdpi.com/article/10.3390/nano15070515/s1, Figure S1: conductivity titration test of the CNFs; Figure S2: TEM image of CNF; Figure S3: static stability of emulsions prepared with different CO:CNF ratios; Figure S4: the tensile strength (a) and elongation (b) at break of derived films; Figure S5: the surface tension of emulsions with different sophorolipids concentrations.

## References

[1] FAO FAO/Foodsecurity Report. https://media.un.org/unifeed/en/asset/d323/d3239510.

[2] Réhault-Godbert S., Guyot N., Nys Y. (2019). The golden egg: Nutritional value, bioactivities, and emerging benefits for human health. Nutrients.

[3] Lima H., Souza L. (2018). Vitamin A in the diet of laying hens: Enrichment of table eggs to prevent nutritional deficiencies in humans. World’s Poult. Sci. J..

[4] Wallace T.C., Blusztajn J.K., Caudill M.A., Klatt K.C., Natker E., Zeisel S.H., Zelman K.M. (2018). Choline: The Underconsumed and Underappreciated Essential Nutrient. Nutr. Today.

[5] Sharaf Eddin A., Ibrahim S.A., Tahergorabi R. (2019). Egg quality and safety with an overview of edible coating application for egg preservation. Food Chem..

[6] Zhang Y., Liu K., Zhang Z., Tian S., Liu X., Qi H., Dong D., Wang Y., Liu M., Li X. (2021). A severe gastroenteritis outbreak of Salmonella enterica serovar enteritidis linked to contaminated egg fried rice, China, 2021. Front. Microbiol..

[7] Figueiredo T.C., Assis D.C., Menezes L.D., Oliveira D.D., Lima A.L., Souza M.R., Heneine L.G., Cançado S.V. (2014). Effects of packaging, mineral oil coating, and storage time on biogenic amine levels and internal quality of eggs. Poult. Sci..

[8] da Silva Pires P.G., da Silva Pires P.D., Cardinal K.M., Bavaresco C. (2020). The use of coatings in eggs: A systematic review. Trends Food Sci. Technol..

[9] Peerzada Gh J., Sinclair B.J., Perinbarajan G.K., Dutta R., Shekhawat R., Saikia N., Chidambaram R., Mossa A.-T. (2023). An overview on smart and active edible coatings: Safety and regulations. Eur. Food Res. Technol..

[10] Raghav P.K., Agarwal N., Saini M. (2016). Edible coating of fruits and vegetables: A review. Int. J. Sci. Res. Mod. Edu..

[11] Caner C., Yüceer M. (2015). Efficacy of various protein-based coating on enhancing the shelf life of fresh eggs during storage. Poult. Sci..

[12] Pirsa S., Aghbolagh Sharifi K. (2020). A review of the applications of bioproteins in the preparation of biodegradable films and polymers. J. Chem. Lett..

[13] Coltelli M.-B., Wild F., Bugnicourt E., Cinelli P., Lindner M., Schmid M., Weckel V., Müller K., Rodriguez P., Staebler A. (2016). State of the Art in the Development and Properties of Protein-Based Films and Coatings and Their Applicability to Cellulose Based Products: An Extensive Review. Coatings.

[14] Nongtaodum S., Jangchud A., Jangchud K., Dhamvithee P., No H.K., Prinyawiwatkul W. (2013). Oil Coating Affects Internal Quality and Sensory Acceptance of Selected Attributes of Raw Eggs during Storage. J. Food Sci..

[15] Caner C., Cansiz O. (2007). Effectiveness of chitosan-based coating in improving shelf-life of eggs. J. Sci. Food Agric..

[16] Xie B., Zhang X., Luo X., Wang Y., Li Y., Li B., Liu S. (2020). Edible coating based on beeswax-in-water Pickering emulsion stabilized by cellulose nanofibrils and carboxymethyl chitosan. Food Chem..

[17] Mouzakitis C.-K., Sereti V., Matsakidou A., Kotsiou K., Biliaderis C.G., Lazaridou A. (2022). Physicochemical properties of zein-based edible films and coatings for extending wheat bread shelf life. Food Hydrocoll..

[18] Sun R., Song G., Zhang H., Zhang H., Chi Y., Ma Y., Li H., Bai S., Zhang X. (2021). Effect of basil essential oil and beeswax incorporation on the physical, structural, and antibacterial properties of chitosan emulsion based coating for eggs preservation. LWT.

[19] da Silva Pires P.G., Bavaresco C., da Silva Pires P.D., Cardinal K.M., Leuven A.F.R., Andretta I. (2021). Development of an innovative green coating to reduce egg losses. Clean. Eng. Technol..

[20] Garavand F., Nooshkam M., Khodaei D., Yousefi S., Cacciotti I., Ghasemlou M. (2023). Recent advances in qualitative and quantitative characterization of nanocellulose-reinforced nanocomposites: A review. Adv. Colloid Interface Sci..

[21] Phanthong P., Reubroycharoen P., Hao X., Xu G., Abudula A., Guan G. (2018). Nanocellulose: Extraction and application. Carbon Resour. Convers..

[22] Yin R., Yang S., Li Q., Zhang S., Liu H., Han J., Liu C., Shen C. (2020). Flexible conductive Ag nanowire/cellulose nanofibril hybrid nanopaper for strain and temperature sensing applications. Sci. Bull..

[23] Dickinson E. (2003). Hydrocolloids at interfaces and the influence on the properties of dispersed systems. Food Hydrocoll..

[24] Deen A., Visvanathan R., Wickramarachchi D., Marikkar N., Nammi S., Jayawardana B.C., Liyanage R. (2021). Chemical composition and health benefits of coconut oil: An overview. J. Sci. Food Agric..

[25] Cho W.Y., Ng J.F., Yap W.H., Goh B.H. (2022). Sophorolipids—Bio-Based Antimicrobial Formulating Agents for Applications in Food and Health. Molecules.

[26] Wang D., Xiao H., Lyu X., Chen H., Wei F. (2023). Lipid oxidation in food science and nutritional health: A comprehensive review. Oil Crop Sci..

[27] Arora J., Ranjan A., Chauhan A., Biswas R., Rajput V.D., Sushkova S., Mandzhieva S., Minkina T., Jindal T. (2022). Surfactant pollution, an emerging threat to ecosystem: Approaches for effective bacterial degradation. J. Appl. Microbiol..

[28] Chen S., Yue N., Cui M., Penkova A., Huang R., Qi W., He Z., Su R. (2022). Integrating direct reuse and extraction recovery of TEMPO for production of cellulose nanofibrils. Carbohydr. Polym..

[29] Pan D., Li Y., Hu Y., Li R., Gao X., Fan X., Fang H., Du Q., Zhou C. (2023). Effect of different concentrations of gellan gum with/without 0.50% basil essential oil on the physicochemical properties of gellan gum-rice bran oil coating emulsions and their application in egg preservation. Food Chem..

[30] (2006). Plastics. Determination of tensile properties. Part 3: Test conditions for films and sheets.

[31] (2021). Test method for water vapor transmission of plastic film and sheet—Desiccant method and water method.

[32] Deng Z., Jung J., Simonsen J., Zhao Y. (2017). Cellulose nanomaterials emulsion coatings for controlling physiological activity, modifying surface morphology, and enhancing storability of postharvest bananas (*Musa acuminate*). Food Chem..

[33] Moats W. (1978). Egg washing—A review. J. Food Prot..

[34] Wang L., Chen C., Wang J., Gardner D.J., Tajvidi M. (2020). Cellulose nanofibrils versus cellulose nanocrystals: Comparison of performance in flexible multilayer films for packaging applications. Food Packag. Shelf Life.

[35] Cano-Sarmiento C., Téllez-Medina D.I., Viveros-Contreras R., Cornejo-Mazón M., Figueroa-Hernández C.Y., García-Armenta E., Alamilla-Beltrán L., García H.S., Gutiérrez-López G.F. (2018). Zeta Potential of Food Matrices. Food Eng. Rev..

[36] Goodarzi F., Zendehboudi S. (2019). A comprehensive review on emulsions and emulsion stability in chemical and energy industries. Can. J. Chem. Eng..

[37] He Y., Wang C., Liu Y., Chen J., Wei Y., Chen G. (2024). Pickering emulsions stabilized by cellulose nanofibers with tunable surface properties for thermal energy storage. Int. J. Biol. Macromol..

[38] Bhasney S.M., Patwa R., Kumar A., Katiyar V. (2017). Plasticizing effect of coconut oil on morphological, mechanical, thermal, rheological, barrier, and optical properties of poly(lactic acid): A promising candidate for food packaging. J. Appl. Polym. Sci..

[39] Fan Z., Hao Y., Wang Y., Hu X., Li T. (2023). Characterisation of hyaluronic acid-curcumin-cellulose nanofibre composite film and application in egg preservation. Int. J. Food Sci. Technol..

[40] Irfan M., Gading B.M.W.T., Sukoco H., Mukhlisah A.N., Maruddin F., Prahesti K.I. (2023). Weight loss and yolk index of commercial chicken eggs as affected by sappan wood extract (*Caesalpinia sappan* L.) and storage time. AIP Conf. Proc..

[41] Wang J., Gardner D.J., Stark N.M., Bousfield D.W., Tajvidi M., Cai Z. (2018). Moisture and oxygen barrier properties of cellulose nanomaterial-based films. ACS Sustain. Chem. Eng..

[42] Zinke A., Pottackal N., Zahin F., Nur M.I., Ahmed F., Ji Y., Mohammed Z., Meyer M.D., Miller C., Bennett M.R. (2024). Preserving Fresh Eggs via Egg-Derived Bionanocomposite Coating. Adv. Funct. Mater..

[43] Fernández-Saiz P., Lagaron J.M. (2011). Chitosan for Film and Coating Applications. Biopolymers—New Materials for Sustainable Films and Coatings.

[44] Galus S., Kadzińska J. (2016). Moisture Sensitivity, Optical, Mechanical and Structural Properties of Whey Protein-Based Edible Films Incorporated with Rapeseed Oil. Food Technol. Biotechnol..

[45] Casariego A., Souza B.W.S., Vicente A.A., Teixeira J.A., Cruz L., Díaz R. (2008). Chitosan coating surface properties as affected by plasticizer, surfactant and polymer concentrations in relation to the surface properties of tomato and carrot. Food Hydrocoll..

[46] Yuceer M., Caner C. (2014). Antimicrobial lysozyme–chitosan coatings affect functional properties and shelf life of chicken eggs during storage. J. Sci. Food Agric..

[47] Keener K.M., Anderson K.E., Curtis P.A., Foegeding J.B. (2004). Determination of Cooling Rates and Carbon Dioxide Uptake in Commercially Processed Shell Eggs Using Cryogenic Carbon Dioxide Gas12. Poult. Sci..

[48] Xu L., Zhang H., Lv X., Chi Y., Wu Y., Shao H. (2016). Internal quality of coated eggs with soy protein isolate and montmorillonite: Effects of storage conditions. Int. J. Food Prop..

