# Supplementary material for: Sustainable Cellulose Nanofibril−Stabilized Pickering Emulsions for Fresh Egg Preservation
Source: Nanomaterials (Basel). 2025 Mar 29;15(7):515. doi: 10.3390/nano15070515 (PMC11990888; doi:10.3390/nano15070515)
Supplement: Supplementary file 1 [file nanomaterials-15-00515-s001.zip › nanomaterials-3529926-supplementary.pdf]

Supporting information

Sustainable cellulose nanofibril–stabilized Pickering emulsions for fresh egg preservation

Hao Li [1,†], Lei Zhang [1,†], Mei Cui [1,*], Renliang Huang [2,*], Rongxin Su [1,2,3]

[1] State Key Laboratory of Chemical Engineering, Tianjin Key Laboratory of Membrane Science and Desalination Technology, School of Chemical Engineering and Technology, Tianjin University, Tianjin, 300072, PR China

[2] Tianjin Key Laboratory for Marine Environmental Research and Service, School of Marine Science and Technology, Tianjin University, Tianjin, 300072, PR China

[3] Zhejiang Institute of Tianjin University, Ningbo, Zhejiang, 315201, China

[†] These authors contributed equally to this work.

* Correspondence: meicui@tju.edu.cn; tjuhrl@tju.edu.cn

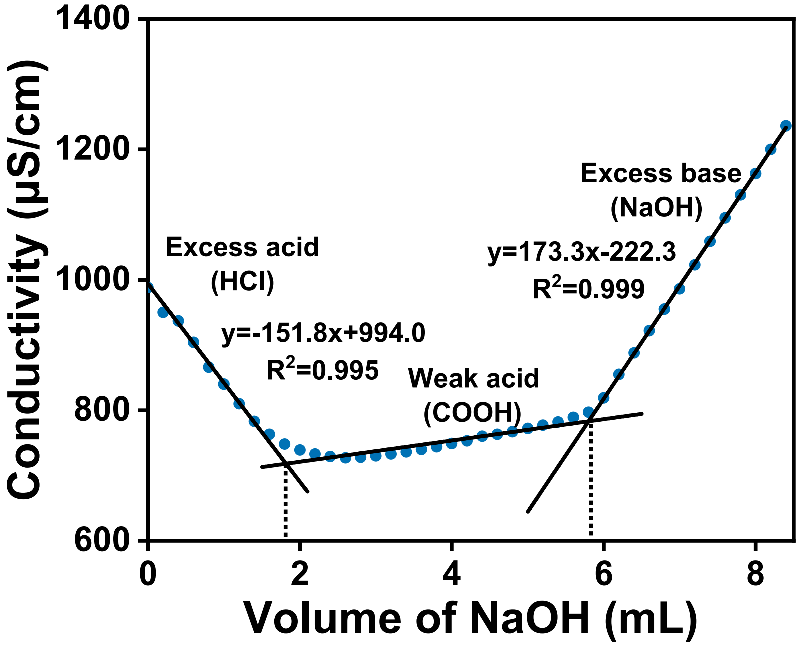

Figure S1. Conductivity titration test of the CNFs.

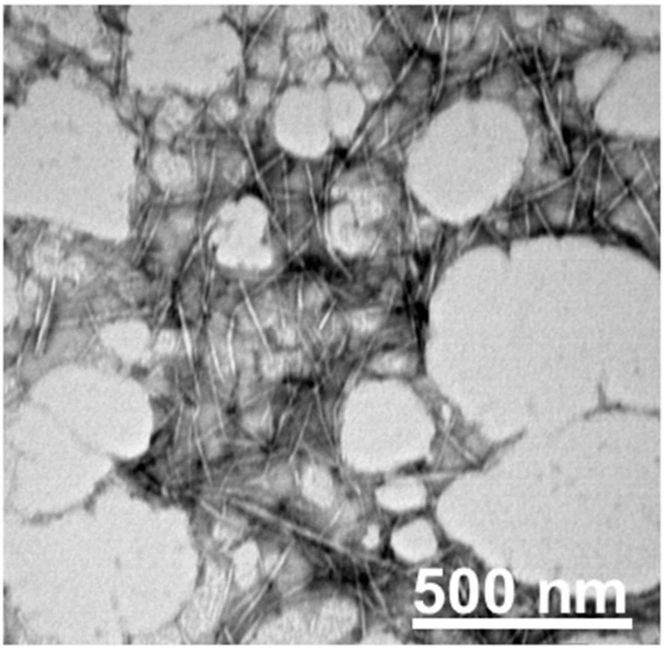

Figure S2. TEM image of CNF.

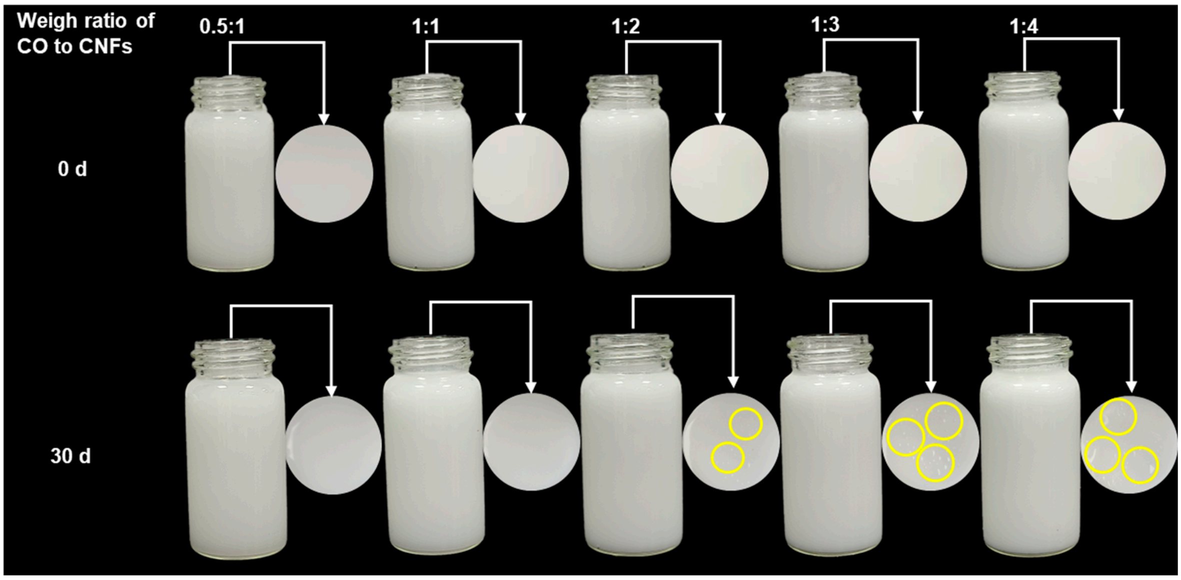

Figure S3. Static stability of emulsions prepared with different CO:CNF ratios.

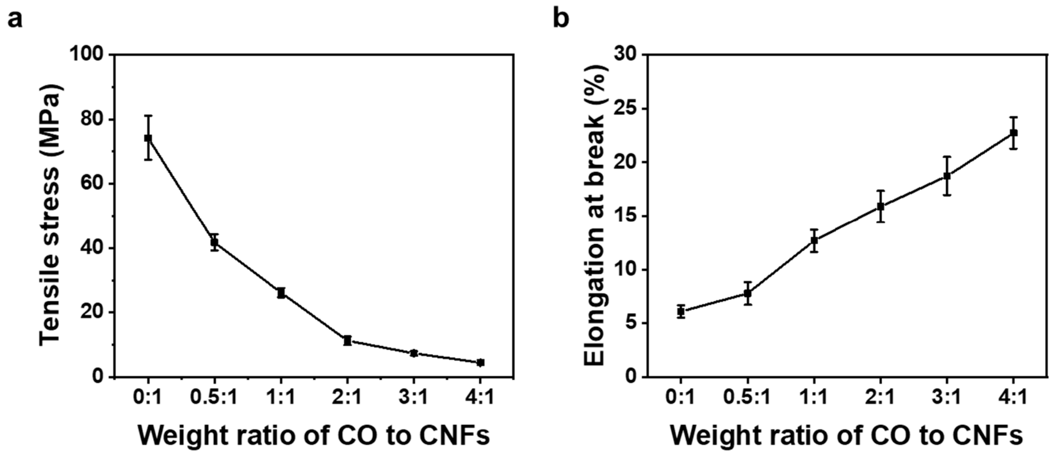

Figure S4. The tensile strength (a) and elongation (b) at break of derived films.

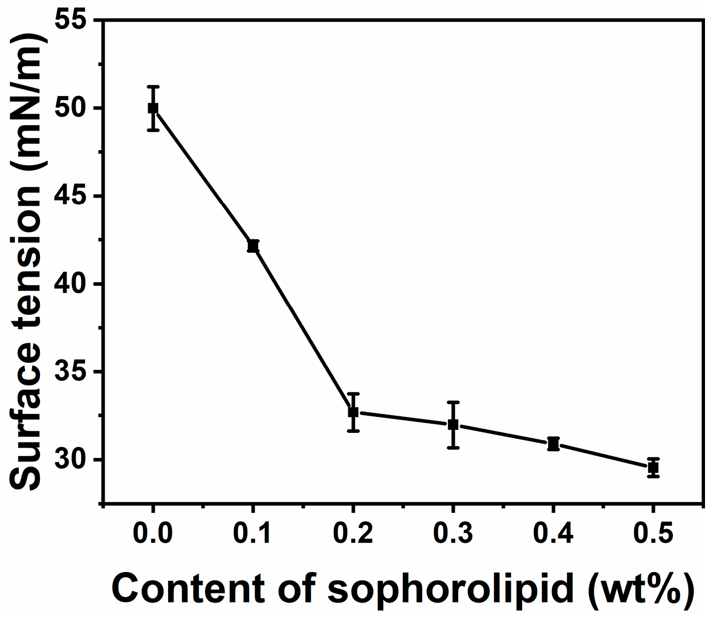

Figure S5. The surface tension of emulsions with different sophorolipid concentrations.
